# Illusory patterns are fishy for fish, too

**DOI:** 10.3389/fncir.2013.00137

**Published:** 2013-08-28

**Authors:** Christian Agrillo, Maria Elena Miletto Petrazzini, Marco Dadda

**Affiliations:** Department of General Psychology, University of PadovaPadova, Italy

**Keywords:** visual illusion, comparative perception, fish, illusory pattern, animal models

It has been widely recognized that size, shape, and distance perception are not the mere translation of images in the eyes, as retinal images are inherently ambiguous. Some form of knowledge and/or assumptions by unconscious inductive inference seems to be necessary (Gregory, 1997). With respect to this topic, visual illusions are a valuable tool for understanding the neuro-cognitive systems underlying visual perception by indirectly revealing the hidden constraints of the perceptual system in a way that normal perception cannot. In humans, such constraints have been often summarized as the so-called “Gestalt principles,” which can be briefly described by the motto “the whole is greater than the sum of its parts” (Wertheimer, 1938). Almost a century of experimental investigation on visual illusions has broadened our comprehension of the perceptual mechanisms that enable us to perceive figures and forms instead of just a collection of lines and curves. Such mechanisms are highly adaptive, as they allow for a quick and stable picture of the environment, enabling an appropriate motor response in every context (Ikin and Turner, 1972).

Given their high ecological value, there is little reason to believe that selective pressures to develop a visual system that is able to segregate objects from the background have acted only on hominids. Indeed, over the last decade, research has demonstrated that both apes and monkeys are deceived by illusory patterns. For instance, baboons perceive the Zöllner illusion (Benhar and Samuel, 1982), capuchin monkeys perceive the Müller-Lyer illusion (Suganuma et al., 2007), and rhesus monkeys perceive numerosity illusion (Beran, 2006; Beran and Parrish, 2013), thus showing that the organization of visual information is similar between human and non-human primates.

Despite the existence of a large number of studies, it is still unclear to what extent previous experience plays a role in how the brain/mind interprets and reconstructs physical reality (Hebb, 1949; Bod, 2002; Quinn and Bhatt, 2006). For practical and ethical reasons, it is very difficult to manipulate experiences during developmental periods in human and non-human primates. Furthermore, as primates lack independence at birth, different procedures are used for studying newborns, juveniles, or adults, presenting one of the major drawbacks when studying the development of visual perception in primates, i.e., the difficulty of devising experimental paradigms applicable to different ages (Bisazza et al., 2010). The recent discovery that even relatively simple organisms like fish, whose divergence seemingly occurred approximately 450 million years ago (Kumar and Hedges, 1998), also perceive visual illusions, as humans do, paves the way for the use of new animal models to investigate the relative contribution of genes and experience.

Redtail splitfin, for instance, was shown to be able to perceive illusory contours (Sovrano and Bisazza, 2009). Fish were required to discriminate between a square or a triangle and the corresponding background. After reaching a learning criterion, subjects performed test trials in the presence of two stimuli: one consisted of a subjective figure (triangle or square) induced by interruption or spatial phase-shift of diagonal lines; the other consisted of a series of diagonal lines only. In a subsequent test, two figures were presented: one in which pacmen were positioned in order to reproduce the Kanizsa triangle or square, and one in which the same pacmen were scrambled in different positions so as to prevent an impression of a subjective figure. Discrimination of orientation, rather than discrimination of shape, was also tested in a second experiment. Subjects were initially trained to discriminate between a vertical and a horizontal line with real physical contours. In test trials vertically and horizontally oriented illusory lines were presented, created either through interruption or spatial phase-shift of diagonal lines (see Table 1). Redtail splitfin were found to perceive illusory contours in both experiments.

**Table 1:** **Summary of static illusory patterns investigated in teleost fish (chronological order)**.

**Authors**	**Species**	**Type of illusion**	**Schematic representation of stimuli**
Wyzisk and Neumeyer, 2007	*Carassius auratus*	Illusory contours	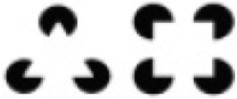
Sovrano and Bisazza, 2008	*Xenotoca eiseni*	Amodal completion	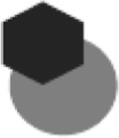
Sovrano and Bisazza, 2009	*Xenotoca eiseni*	Illusory contours	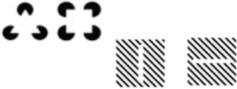
Darmaillacq et al., 2011	*Variola louti* and *Scarus niger*	Amodal completion	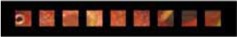

Wyzisk and Neumeyer (2007) successfully trained goldfish to discriminate between triangles and squares. After reaching the learning criterion, the authors presented a Kanizsa triangle and a Kanizsa square, and found that goldfish were able to discriminate between the two patterns based on the illusory contours. Goldfish showed high orientation sensitivity with respect to the pacmen generating the illusory patterns. Interestingly, if black lines were over-imposed on a Kanizsa triangle or square, the illusory perception was disrupted, as has also been reported in humans, suggesting the existence of an end-stopped property similar to the neurons in V2 found in monkeys (von der Heydt, 2004).

Data collected on redtail splitfin and goldfish are particularly interesting as the two species are only distantly related. According to recent estimates, the Ostariophysi, the group to which redtail splitfin belong, and the Acanthopterygii, the group to which goldfish belong, diverged more than 250 million years ago (Steinke et al., 2006). The fact that even distantly related species perceive illusory contours suggests the existence of orientation-selective neurons—responding to edges, lines, or bars of high contrast—in a wide range of teleost fish. Also, more recent evidence further suggests similar perceptual mechanisms between fish and primates: reef fish tested in their natural environment exhibited amodal completion, as they tried to attack their own mirror image even when they could see a fragmented image of themselves (Darmaillacq et al., 2011). It is interesting to note that fish did not attack their imagine when they could see only a portion of the body in a single square, thus showing that their aggressive behavior was not simply triggered by some specific body features, such as color. Amodal completion was also reported in another fish species, the redtail splitfin (Sovrano and Bisazza, 2008).

These studies have theoretical implications in the debate surrounding human visual perception. It has been suggested that a single unit-formation process may underlie modal (the perception of both real and subjective contours) and amodal completion, as completion processes would depend on a common underlying mechanism connecting edges across gaps (Kellman et al., 1998; Palmer, 1999). Fish species reported in the literature (Table 1) showed a successful perception of both modal and amodal completion. This finding indirectly aligns with the idea of a single mechanism for the two processes. Nonetheless, we believe that future research on newborn and juvenile fish will provide even more useful insights, especially in the debate surrounding the developmental trajectories of Gestalt principles. Due to their relatively short lifespan and independence at birth, fish represent an excellent experimental model for studying the development of perception and cognition. Indeed, recent studies have already adopted fish to study the ontogeny and the developmental trajectories of perceptual and cognitive systems (Bisazza et al., 2010; Miletto Petrazzini et al., 2013). Given that adult fish vision seems to be based on Gestalt principles, the development of such principles may be now investigated using newborn/juvenile fish as a model.

A validated method exists to study cognition and perception in newborn fish (Miletto Petrazzini et al., 2012). This method involves introducing two stimuli (i.e., two different geometric figures) at the opposite ends of the tank and delivering food near the discriminative stimulus. Discrimination is inferred from the portion of time spent near the trained stimulus during final probe trials. The method has been shown to be very rapid (only 12 reinforced trials) and successful in discrimination tasks (i.e., circle vs. triangle), thus making it a good candidate for investigating the ontogeny of Gestalt principles in rapidly growing species, such as fish. Based on previous literature, the focus should be given initially to illusory patterns called “Fictions”—including illusory contours—in the classification advanced by Gregory (1997). First, it would be interesting to see if/which Gestalt principles are inherent; if not, it would be challenging to study their developmental trajectory and the influence of maturation and experience.

The use of zebrafish, one of the main model organisms for neurobiology studies of vision and neurodevelopmental genetics, is especially welcome, given the possibility to extend the investigation on illusory perception with genetic and neuroanatomic aspects. The anatomical, physiological, and genetic components of the zebrafish visual system have been widely investigated in both larval and adult individuals (e.g., Bilotta and Saszik, 2001). Several studies indicate that zebrafish are capable of high-level motion processing. In particular, two visually guided behaviors received great attention in the literature: the optokinetic response (OKR) and the optomotor response (OMR). The OKR is a consistent behavior in which moving objects across the visual field evoke stereotyped eye movements (Neuhauss, 2003; Huang and Neuhauss, 2008). These eye movements consist of two distinct components: a smooth pursuit movement and a fast saccade which resets the eyes once the object has left the visual field (Portugues and Engert, 2009). A small hindbrain area in rhombomere 5 has been found to be necessary for this response to occur properly (Schoonheim et al., 2010). Neuhauss et al. (1999) found that zebrafish mutant *belladonna* (*bel*) often displays an OKR opposite to the direction of movement of the objects. Interestingly, Huang et al. (2009) found that a subset of the same mutants also display atypical circular swimming patterns (“looping”) as a result of illusionary self-motion perception. On the other hand, the OMR occurs when a whole-field moving stimulus is presented and the fish turn and swim according to the perceived motion direction (Neuhauss et al., 1999; Portugues and Engert, 2009). Mutants with visual defects—such as the *lakritz*(*lak*) mutant, which lacks a large subset of retinal ganglion cells—fail at the OMR test (Baier, 2000).

In humans, both OKR and OMR have been hypothesized to be involved in different visual illusions (Schor et al., 1984; Riecke et al., 2009). In this sense, the use of mutant zebrafish with opposite OKR, or lacking OMR, will play a key role in verifying the influence of both neural mechanisms in the perception of illusory patterns in a way that is not possible with primates.

Small brains are likely to provide important insights with respect to the ancient philosophical question of how the visual system builds our reality.

## References

[B1] Baier H. (2000). Zebrafish on the move: towards a behavior–genetic analysis of vertebrate vision. Curr. Opin. Neurobiol. 10, 451–455 10.1016/S0959-4388(00)00116-1 10981613

[B2] Benhar E. Samuel D. (1982). Visual illusions in the baboon (*Papio anibis*). Anim. Learn. Behav. 10, 115–118 10.3758/BF03212056

[B3] Beran M. J. (2006). Quantity perception by adult humans (*Homo sapiens*), chimpanzees (*Pan troglodytes*), and rhesus macaques (*Macaca mulatta*) as a function of stimulus organization. Int. J. Comp. Psych. 19, 386–397

[B4] Beran M. J. Parrish A. E. (2013). Visual nesting of stimuli affects rhesus monkeys' (*Macaca mulatta*) quantity judgments in a bisection task. Atten. Percept. Psychophys. 75, 1243–1251 10.3758/s13414-013-0474-5 23709063 PMC3735653

[B5] Bilotta J. Saszik S. (2001). The zebrafish as a model visual system. Int. J. Dev. Neurosci. 19, 621–629 10.1016/S0736-5748(01)00050-8 11705666

[B6] Bisazza A. Piffer L. Serena G. Agrillo C. (2010). Ontogeny of numerical abilities in fish. PLoS ONE 5:e15516 10.1371/journal.pone.0015516 21124802 PMC2991364

[B7] Bod R. (2002). Memory-based models of melodic analysis: challenging the gestalt principles. J. N. Mus. Res. 31, 27–37 10.1076/jnmr.31.1.27.8106

[B8] Darmaillacq A. S. Dickel L. Rahmani N. Shashar N. (2011). Do reef fish, *Variola louti* and Scarus niger, perform amodal completion? evidence from a field study. J. Comp. Psychol. 125, 273–277 10.1037/a0024295 21842982

[B9] Gregory R. (1997). Visual illusions classified. Trends Cogn. Sci. 1, 190–194 10.1016/S1364-6613(97)01060-7 21223901

[B10] Hebb D. O. (1949). The Organization of Behavior. New York, NY: John Wiley.

[B11] Huang Y. Y. Neuhauss S. C. (2008). The optokinetic response in zebrafish and its applications. Front. Biosci. 13, 1899–1916 17981678 10.2741/2810

[B12] Huang Y. Y. Tschopp M. Neuhauss S. C. (2009). Illusionary self-motion perception in zebrafish. PLoS ONE 4:e6550 10.1371/journal.pone.0006550 19672291 PMC2717804

[B13] Ikin M. Turner J. R. G. (1972). Experiments on mimicry: gestalt perception and the evolution of genetic linkage. Nature 239, 525–527 10.1038/239525b0

[B14] Kellman P. J. Yin C. Shipley T. F. (1998). A common mechanism for illusory and occluded object completion. J. Exp. Psych. Hum. Percept. Perform. 24, 859–869 10.1037/0096-1523.24.3.859 9627421

[B15] Kumar S. Hedges S. B. (1998). A molecular timescale for vertebrate evolution. Nature 392, 917–920 10.1038/31927 9582070

[B16] Miletto Petrazzini M. E. Agrillo C. Piffer L. Bisazza A. (2013). Ontogeny of the capacity to compare discrete quantities in fish. Dev. Psychobiol. [Epub ahead of print]. 10.1002/dev.21122 23775761

[B17] Miletto Petrazzini M. E. Agrillo C. Piffer L. Dadda M. Bisazza A. (2012). Development and application of a new method to investigate cognition in newborn guppies. Behav. Brain Res. 233, 443–449 10.1016/j.bbr.2012.05.044 22677276

[B18] Neuhauss S. C. Biehlmaier O. Seeliger M. W. Das T. Kohler K. Harris W. A. (1999). Genetic disorders of vision revealed by a behavioral screen of 400 essential loci in zebrafish. J. Neurosci. 19, 8603–8615 10493760 10.1523/JNEUROSCI.19-19-08603.1999 PMC6783047

[B19] Neuhauss S. C. (2003). Behavioral genetic approaches to visual system development and function in zebrafish. Dev. Neurobiol. 54, 148–160 10.1002/neu.10165 12486702

[B19a] Palmer S. E. (1999). Organizing objects and scenes, in “Vision Science: Photons to Phenomenology,” ed Palmer S E (Cambridge, MA: MIT Press), 254–310

[B20] Portugues R. Engert F. (2009). The neural basis of visual behaviors in the larval zebrafish. Curr. Opin. Neurobiol. 19, 644–647 19896836 10.1016/j.conb.2009.10.007 PMC4524571

[B20a] Quinn P. C. Bhatt R. S. (2006). Are some Gestalt principles deployed more readily than others during early development? The case of lightness versus form similarity. J. Exp. Psych. Hum. Percept. Perform. 32, 1221–1230 10.1037/0096-1523.32.5.1221 17002533

[B21] Riecke B. E. Valjamae A. Schulte-Pelkum J. (2009). Moving sounds enhance the visually-induced self-motion illusion (circular vection) in virtual reality. ACM Trans. Appl. Percept. 6, 7–27 10.1145/1498700.1498701

[B22] Schoonheim P. J. Arrenberg A. B. Del Bene F. Baier H. (2010). Optogenetic localization and genetic perturbation of saccade-generating neurons in zebrafish. J. Neurosci. 30, 7111–7120 10.1523/JNEUROSCI.5193-09.2010 20484654 PMC3842466

[B23] Schor C. M. Lakshminarayanan V. Narayan V. (1984). Optokinetic and vection responses to apparent motion in man. Vis. Res. 24, 1181–1187 10.1016/0042-6989(84)90173-1 6523741

[B24] Sovrano V. A. Bisazza A. (2008). Recognition of partly occluded objects by fish. Anim. Cogn. 11, 161–166 10.1007/s10071-007-0100-9 17636365

[B25] Sovrano V. A. Bisazza A. (2009). Perception of subjective contours in fish. Perception 38, 579–590 10.1068/p6121 19522325

[B26] Steinke D. Salzburger W. Meyer A. (2006). Novel relationships among ten fish model species revealed based on a phylogenomic analysis using ESTs. J. Mol. Evol. 62, 772–784 10.1007/s00239-005-0170-8 16752215

[B27] Suganuma E. Pessoa V. Mongefuentes B. Castro B. Tavares M. (2007). Perception of the Müller-Lyer illusion in capuchin monkeys (*Cebus apella*). Behav. Brain Res. 182, 67–72 10.1016/j.bbr.2007.05.014 17586063

[B28] von der Heydt R. (2004). Image parsing mechanisms of the visual cortex, in The Visual Neurosciences, eds Chalupa L. M. Werner J. S. (Cambridge, MA: MIT Press), 1139–1150

[B29] Wertheimer M. (1938). Laws of organization in perceptual forms, in A Sourcebook of Gestalt Psychology, ed Ellis W. B. (Harcourt: Brace and Company), 71–88

[B30] Wyzisk K. Neumeyer C. (2007). Perception of illusory surfaces and contours in goldfish. Vis. Neurosci. 24, 291–298 10.1017/S095252380707023X 17822573

